# Supplementary material for: Causal Relationship between Aging and Anorexia Nervosa: A White-Matter-Microstructure-Mediated Mendelian Randomization Analysis
Source: Biomedicines. 2024 Aug 16;12(8):1874. doi: 10.3390/biomedicines12081874 (PMC11351342; doi:10.3390/biomedicines12081874)
Supplement: Supplementary file 1 [file biomedicines-12-01874-s001.zip › Supplementary Figure/Figure S8/Leaveoneout plot of MR analysis for TL to ubm-b-2014.pdf]

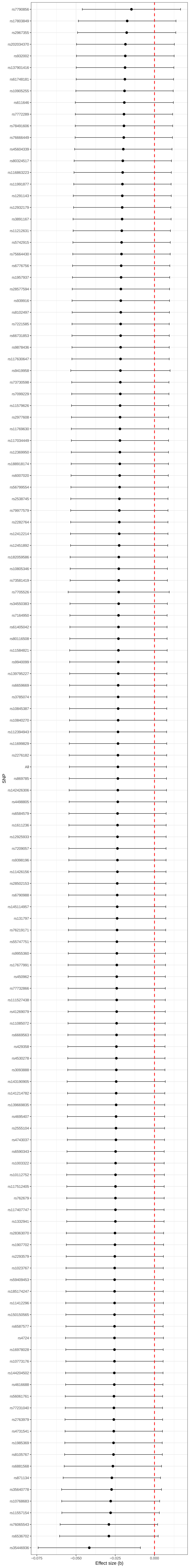

rs7790856
rs17803849
rs2967355
rs202034370
rs932002
rs137901416
rs61748181
rs10905255
rs611646
rs7772289
rs78491606
rs76666449
rs45604339
rs80324517
rs116863223
rs11991877
rs1291143
rs12932179
rs3891167
rs11212631
rs5742915
rs75664430
rs6776756
rs1957937
rs28577594
rs939916
rs8102497
rs7221585
rs66731853
rs9878436
rs117630647
rs9419958
rs73730598
rs7099229
rs11579626
rs2977608
rs11769630
rs117034449
rs12369950
rs188918174
rs6007020
rs56799554
rs2538745
rs79977579
rs2282764
rs12412214
rs12451892
rs182059586
rs10805346
rs73581419
rs7705526
rs34550383
rs7164950
rs61405042
rs80116508
rs11584821
rs9940099
rs139795227
rs6659669
rs3785074
rs10845387
rs10840270
rs112394943
rs11699829
rs2276182
All
rs869785
rs142426306
rs4498805
rs6584579
rs1611236
rs12925933
rs7209057
rs9398196
rs11426156
rs28502153
rs6790988
rs145114957
rs131797
rs76219171
rs55747751
rs9955360
rs17677991
rs450962
rs77732866
rs111527438
rs41269079
rs11085072
rs6669563
rs429358
rs4530278
rs3093888
rs143190905
rs141214782
rs139669835
rs4695407
rs2555104
rs4743037
rs6590343
rs1003322
rs10112752
rs117512405
rs762679
rs117407747
rs1332941
rs28363070
rs1907702
rs2293579
rs1023767
rs59409453
rs185174247
rs11412296
rs150150565
rs6587577
rs4724
rs16978028
rs10773176
rs144204502
rs4616688
rs56061761
rs77231040
rs2763979
rs4731541
rs1985369
rs8105767
rs6881568
rs871134
rs35640778
rs10768683
rs11557154
rs76065543
rs6536702
rs35446936
SNP
−0.075
−0.050
−0.025
0.000
Effect size (b)
